# Perinatal and Early Respiratory Factors Associated with Invasive Mechanical Ventilation in Very Preterm Infants: A Retrospective Cohort Study from Kazakhstan

**DOI:** 10.3390/jcm15207879

**Published:** 2026-10-13

**Authors:** Umid Alimukhamedov, Nishankul Bozhbanbayeva, Merih Çetinkaya, Talgat Tolykbayev, Altynay Sadykova, Bekzat Suieubekov, Ulykpan Alseitov

**Affiliations:** 1 Department of Neonatology, Asfendiyarov Kazakh National Medical University, Almaty 050012, Kazakhstan; bozhbanbaeva.n@kaznmu.kz; 2 JSC “Scientific Center of Obstetrics, Gynecology and Perinatology”, Almaty 050020, Kazakhstan; 3 Division of Neonatology, Department of Pediatrics, Faculty of Medicine, Istanbul Atlas University, Istanbul 34408, Turkey; merih.cetinkaya@atlas.edu.tr; 4 Zhambyl Regional Perinatal Center, Department of Health of the Akimat of Zhambyl Region, Taraz 080000, Kazakhstan; 5 Department of Children’s Diseases Named After Professor N.A. Barlybayeva, Asfendiyarov Kazakh National Medical University, Almaty 050012, Kazakhstan; 6 Department of Pediatric Anesthesiology and Intensive Care, Asfendiyarov Kazakh National Medical University, Almaty 050012, Kazakhstan; 7 Children’s Emergency Medicine Center, Almaty 050040, Kazakhstan

**Keywords:** very preterm infant, invasive mechanical ventilation, CPAP, respiratory distress syndrome, LISA

## Abstract

**Background**: Evidence on factors associated with the timing of invasive mechanical ventilation (IMV) among very preterm infants in Central Asia remains limited. **Methods**: This retrospective cohort study included infants born at 22–30 weeks gestation in two tertiary perinatal centers in Kazakhstan between 2021 and 2025. Analyses were restricted to 381 infants with complete data. IMV initiated during the first week of life was classified as early IMV, and IMV initiated after the first week as late IMV. Separate multivariable logistic regression models were constructed for any, early, and late IMV. **Results**: Any IMV was recorded in 191 infants (50.1%), including 132 (34.6%) with early IMV and 59 (15.5%) with late IMV. Early IMV was associated with gestational age ≤ 27 weeks (aOR 3.91, 95% CI 1.66–9.22), absence of spontaneous breathing at birth (aOR 151.93, 95% CI 18.26–1263.88), and initial FiO_2_ > 0.30 (aOR 4.39, 95% CI 1.97–9.77). Complete antenatal corticosteroid exposure was inversely associated with early IMV (aOR 0.38, 95% CI 0.20–0.72). Late IMV was associated with birth weight < 1000 g (aOR 3.51, 95% CI 1.47–8.35) and initial FiO_2_ > 0.30 (aOR 2.12, 95% CI 1.02–4.39). An initial set CPAP ≥ 12 cmH_2_O was recorded in 71.9% of infants and was associated with any IMV but not with early or late IMV separately. **Conclusions**: Early and late IMV were associated with different clinical patterns. These observational associations may support risk assessment but do not establish causal effects of respiratory-support practices.

## 1. Introduction

Respiratory distress syndrome remains one of the principal causes of morbidity and mortality among very preterm infants. Structural lung immaturity, surfactant deficiency, and limited respiratory reserve frequently necessitate respiratory support immediately after birth. Although invasive mechanical ventilation (IMV) can be lifesaving, exposure to positive-pressure ventilation may contribute to ventilator-induced lung injury and the subsequent development of bronchopulmonary dysplasia (BPD). Current European recommendations therefore emphasize comprehensive perinatal lung-protective care, including antenatal corticosteroid administration, delayed cord clamping, support of spontaneous breathing, early continuous positive airway pressure (CPAP), controlled oxygen supplementation, and selective surfactant therapy [1].

Less invasive surfactant administration (LISA) has become an important component of this strategy for spontaneously breathing preterm infants receiving CPAP [2]. By delivering surfactant through a thin tracheal catheter without conventional endotracheal ventilation, LISA aims to preserve spontaneous breathing and reduce exposure to IMV. A Cochrane review found that thin-catheter surfactant administration was associated with lower risks of intubation within the first 72 h, death or BPD, and in-hospital mortality compared with surfactant administration through an endotracheal tube [3]. However, the OPTIMIST-A randomized clinical trial did not demonstrate a statistically significant reduction in its primary composite outcome of death or BPD, highlighting the heterogeneity of treatment effects across different gestational-age groups and clinical settings [4].

Despite the increasing use of noninvasive respiratory support and LISA, a substantial proportion of very preterm infants still require IMV. Previous studies have identified lower gestational age, lower birth weight, absent or incomplete antenatal corticosteroid exposure, higher early oxygen requirement, and greater initial respiratory instability as factors associated with failure of CPAP or LISA [5,6,7,8,9,10]. Evidence from Asian settings also demonstrates variation in LISA outcomes. A retrospective Chinese study found similar rates of mechanical ventilation within 72 h and BPD after LISA and intubation–surfactant–extubation (INSURE), whereas an Indian randomized trial found a lower rate of CPAP failure with LISA using an infant feeding tube but no reduction in the total duration of respiratory support [11,12]. Neither study distinguished early from late IMV during hospitalization. Nevertheless, definitions of treatment failure vary considerably. Most studies define failure as intubation within 72 h after LISA, whereas clinically relevant IMV may also be initiated later during hospitalization. Furthermore, much of the available evidence originates from European or other high-resource neonatal networks, and data from Central Asian neonatal populations remain limited [13]. Differences in referral pathways, perinatal risk profiles, clinical protocols, and access to respiratory technologies may affect both the use and outcomes of noninvasive stabilization.

Therefore, this study aimed to identify routinely recorded perinatal and early respiratory factors associated with IMV during hospitalization among very preterm infants treated at tertiary perinatal centers in Kazakhstan. Secondary objectives were to evaluate factors associated with death before discharge and the composite outcome of recorded BPD or death, and to determine whether the principal associations remained consistent among infants treated with LISA. We hypothesized that greater immaturity and early respiratory compromise would be associated with higher odds of IMV, whereas complete antenatal corticosteroid exposure would be associated with lower odds of adverse respiratory outcomes.

## 2. Methods

### 2.1. Study Design and Setting

This multicenter retrospective cohort study was conducted at two tertiary perinatal centers in Kazakhstan. The study included infants born between 2021 and 2025 and used routinely collected clinical data on perinatal characteristics, delivery-room stabilization, early respiratory management, and in-hospital outcomes. Treating neonatal teams determined clinical management according to institutional protocols, and the study did not influence it. Reporting followed the STROBE recommendations.

### 2.2. Study Population

Infants were eligible if they were treated at either participating tertiary perinatal center between 2021 and 2025, had a recorded gestational age of 22–30 completed weeks, and received respiratory stabilization in the delivery room or during the early neonatal period.

Eligible infants were included in the complete-case analytical cohort when all variables required for the prespecified analyses were available and could be assigned to the harmonized categories. The variables collected and their definitions are described in Section 2.3.

**Figure 1:**
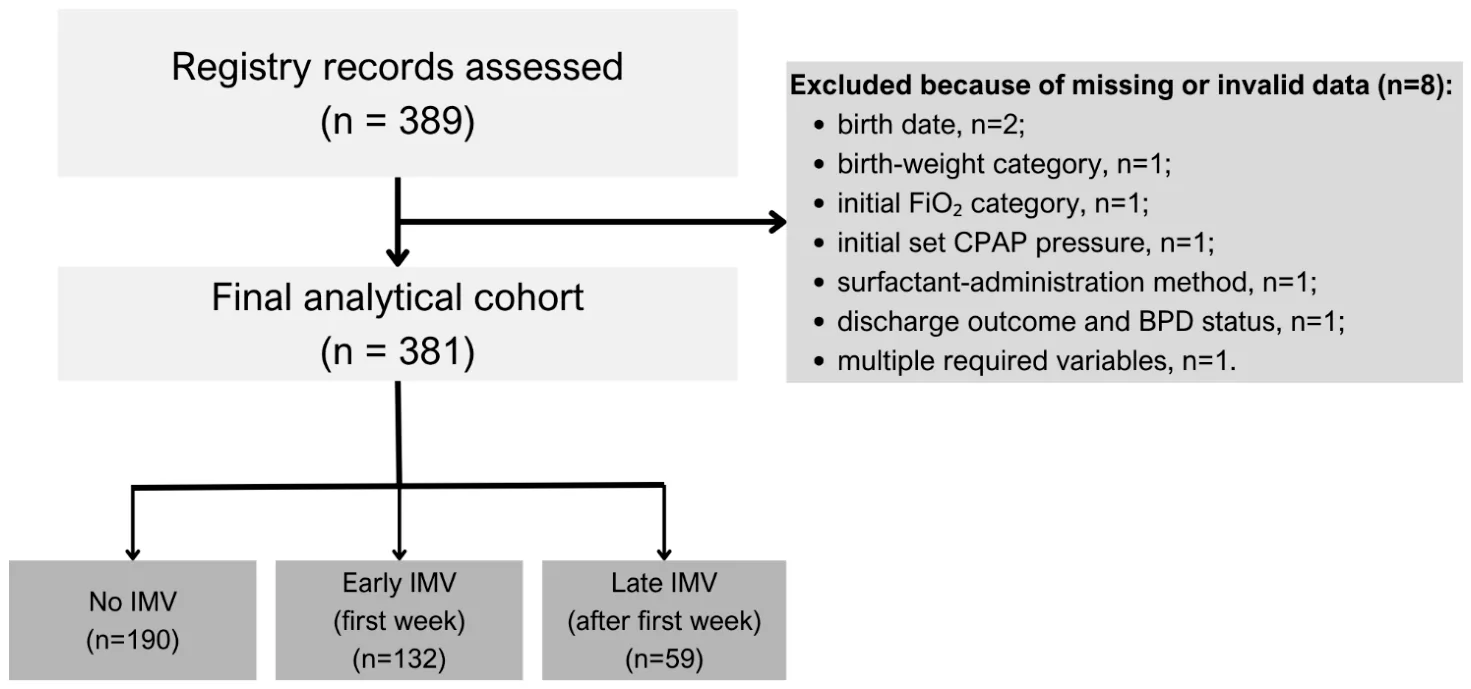
Formation of the complete-case analytical cohort.

Records were excluded if they were duplicated, fell outside the prespecified gestational-age or study-period range, or contained missing, implausible, or noncodable values for any variable required for the complete-case analysis. No duplicate records or records outside the gestational-age range were identified. Eight of 389 potentially eligible records did not meet the complete-case criteria, resulting in a final analytical cohort of 381 infants (Figure 1). Major congenital anomalies were defined as congenital lung malformations, congenital heart defects causing marked hemodynamic disturbance and respiratory impairment, chromosomal abnormalities, or congenital anomalies incompatible with life. Infants with these conditions were excluded.

### 2.3. Data Collection and Variable Definitions

Data were obtained from institutional neonatal registries containing information collected during routine clinical care. Each record represented one infant. Duplicate records were defined as repeated entries with matching infant identifiers and birth dates; after verification, only one record per infant was retained. Extracted variables included birth date, birth-weight category, gestational-age category, antenatal corticosteroid exposure, conception by in vitro fertilization, multiple pregnancy, delayed cord clamping, spontaneous breathing at birth, initial fraction of inspired oxygen (FiO_2_), initial set CPAP, caffeine administration, surfactant-administration method, IMV, length of stay in the neonatal intensive care unit, bronchopulmonary dysplasia (BPD), and status at hospital discharge.

To ensure consistency across the two centers, equivalent registry fields were mapped to common analytical variables, and identical definitions, category thresholds, and permitted numerical codes were applied. The original birth-weight categories of 500–749, 750–999, 1000–1249, and 1250–1499 g were collapsed into <1000 versus ≥1000 g. For the regression analyses, gestational-age categories were collapsed into ≤27 versus 28–30 completed weeks. The original categories of 22–24, 25, 26, 27, 28, 29, and 30 weeks were retained for the descriptive analysis.

Antenatal corticosteroid exposure was classified as complete versus absent or partial. Delayed cord clamping was considered present when a delay of either 30 or 60 s was recorded. Spontaneous breathing at birth was categorized as present or absent. Initial oxygen requirement was classified as FiO_2_ ≤ 0.30 versus >0.30. Initial set CPAP was categorized as <12 versus ≥12 cmH_2_O.

Birth years were grouped into three periods: 2021–2022, 2023, and 2024–2025. Births occurring in 2024 and 2025 were combined because the number of infants recorded in 2024 alone was insufficient for stable period-specific estimates. Surfactant administration was categorized as LISA, intubation–surfactant–extubation (INSURE), or surfactant administration following intubation. LISA was defined as surfactant administration through a thin tracheal catheter while spontaneous breathing and noninvasive respiratory support were maintained. INSURE involved brief endotracheal intubation for surfactant administration followed by planned extubation to noninvasive support.

Data-quality checks included assessment of missing values, verification of permitted category codes, and evaluation of internal consistency between gestational age, birth weight, respiratory status, surfactant administration, and subsequent respiratory support. Implausible values were defined as values outside the permitted categories or internally inconsistent with related variables. These values were checked against the source records and, when they could not be resolved or reliably assigned to a prespecified category, were treated as missing rather than reassigned.

### 2.4. Respiratory Stabilization and Exposure Definitions

Initial CPAP was delivered using heated and humidified gas at a flow rate of 20 L/min through a face mask. The initial FiO_2_ was 0.21 and was subsequently titrated according to preductal oxygen saturation. For the analysis, FiO_2_ > 0.30 represented the recorded oxygen requirement during initial stabilization. The registry did not link this value to a predefined postnatal time point, and serial FiO_2_ measurements were unavailable. The local delivery-room stabilization protocol described a set CPAP of 15 cmH_2_O maintained during the approximately 30–40-min stabilization procedure. After initial stabilization, the face mask could be replaced with a mononasal or nasopharyngeal interface. When indicated, less invasive surfactant administration was performed during ongoing CPAP. The available protocol description did not specify gestational-age-specific pressure adjustments. However, the registry contained initial set CPAPs of 8, 10, 12, and 15 cmH_2_O and did not record the clinical rationale for selecting a particular setting for an individual infant. For the present analysis, initial set CPAP was categorized as <12 or ≥12 cmH_2_O. The registry recorded the initial set CPAP; delivered airway pressure and interface leakage were unavailable.

### 2.5. Outcomes

The primary outcome was receipt of invasive mechanical ventilation (IMV) during hospitalization. The source registry classified infants into three mutually exclusive categories: no IMV, IMV initiated during the first week of life (early IMV), and IMV initiated after the first week of life (late IMV). Early and late IMV were combined to define the binary outcome of any IMV versus no IMV. For the timing-specific analyses, early IMV was compared with no early IMV, whereas the analysis of late IMV excluded infants who received early IMV and compared infants with late IMV with those who never received IMV.

The registry did not capture standardized intubation thresholds or patient-level clinical indications for transitioning from noninvasive respiratory support to IMV. Information on respiratory effort, recurrent apnea, blood-gas parameters, including pH and PaCO_2_, oxygen-saturation trends, hemodynamic status, and response to surfactant therapy at the time of escalation was unavailable. Consequently, the precise clinical decision pathway leading to IMV could not be reconstructed retrospectively, and it was not possible to determine whether infants met uniform physiological criteria for CPAP failure.

Secondary outcomes were death before hospital discharge, BPD as recorded in the neonatal registry, and the composite outcome of BPD or death. Because the exact postmenstrual age at BPD assessment and BPD severity were unavailable, BPD was analyzed according to its recorded binary status. Accordingly, recorded BPD and the composite of recorded BPD or death were treated as exploratory, registry-specific outcomes and were not considered directly comparable with outcomes based on contemporary standardized BPD definitions. An exploratory subgroup analysis was performed among infants for whom LISA was recorded as the surfactant-administration method.

### 2.6. Statistical Analysis

All analyses were restricted to the complete-case cohort of 381 infants, and missing values were not imputed. Categorical variables were summarized as numbers and percentages. Characteristics were compared across the no-IMV, early-IMV, and late-IMV groups using Pearson’s chi-square test or Fisher’s exact test, as appropriate.

Three separate multivariable binary logistic regression models were constructed to estimate adjusted associations with any IMV, early IMV, and late IMV. The any-IMV model compared 191 infants who received early or late IMV with 190 infants who never received IMV. The early-IMV model compared 132 infants who received IMV during the first week of life with 249 infants who did not receive early IMV. The late-IMV model was restricted to 249 infants who did not receive IMV during the first week and compared 59 infants with late IMV with 190 infants who never received IMV.

Candidate predictors were selected a priori according to clinical relevance and availability in the harmonized registries. These included birth weight < 1000 g, gestational age ≤27 weeks, complete antenatal corticosteroid course, delayed cord clamping, absence of spontaneous breathing at birth, initial FiO_2_ > 0.30, initial set CPAP ≥ 12 cmH_2_O, and birth period. Reference categories were birth weight ≥ 1000 g, gestational age 28–30 weeks, absent or partial antenatal corticosteroid exposure, no delayed cord clamping, presence of spontaneous breathing at birth, initial FiO_2_ ≤ 0.30, initial set CPAP < 12 cmH_2_O, and birth period 2021–2022. Birth weight and gestational age were retained simultaneously because they represent clinically distinct dimensions of immaturity. Predictors that could not be reliably estimated because of complete or quasi-complete separation were omitted from the affected model.

Additional exploratory multivariable models examined death before hospital discharge and the composite outcome of BPD or death. In the LISA subgroup analysis, respiratory effort at birth was omitted because spontaneous breathing is an eligibility requirement for LISA. No automated or significance-based variable-selection procedure was used.

Mortality before discharge was additionally summarized according to the original gestational-age categories of 22–24, 25, 26, 27, 28, 29, and 30 weeks. Multicollinearity was assessed using variance inflation factors, with values ≥5 considered potentially problematic. Associations are reported as adjusted odds ratios (aORs) with 95% confidence intervals (CIs). All tests were two-sided. For the prespecified IMV models, *p* < 0.05 was considered statistically significant. Secondary-outcome and subgroup analyses were exploratory; no adjustment for multiple comparisons was performed, and these findings were interpreted as hypothesis-generating.

Although the data originated from two centers, the analytical dataset did not contain a reliable patient-level center identifier. Therefore, center-stratified analyses and adjustment for center were not performed. Statistical analyses were conducted using Python (version 3.12) (Python Software Foundation, Beaverton, OR, USA).

### 2.7. Ethical Considerations

The study was approved by the Local Commission on Bioethics of the Asfendiyarov Kazakh National Medical University (Protocol No. 30 [166], study No. 15-140425, 29 September 2025).

## 3. Results

### 3.1. Cohort Characteristics

Of 389 potentially eligible infant records, 381 (97.9%) had complete data for all variables included in the main analyses and constituted the analytical cohort; eight records (2.1%) were excluded because of missing or invalid data (Figure 1). The cohort included 116 infants (30.4%) born in 2021–2022, 148 (38.8%) born in 2023, and 117 (30.7%) born in 2024–2025.

Among the 381 included infants, 191 (50.1%) received IMV, including 132 (34.6%) with early IMV and 59 (15.5%) with late IMV; 190 infants (49.9%) never received IMV. Compared with the other groups, infants receiving early IMV were more frequently born at ≤27 weeks, had birth weight < 1000 g, lacked spontaneous breathing at birth, and required an initial FiO_2_ >0.30, whereas complete antenatal corticosteroid exposure, delayed cord clamping, and LISA were less frequent (Table 1). An initial set CPAP ≥ 12 cmH_2_O was recorded in 274 infants (71.9%). Its use declined from 100% in 2021–2022 to 80.4% in 2023 and 33.3% in 2024–2025 but did not differ significantly among the no-IMV, early-IMV, and late-IMV groups.

### 3.2. Factors Associated with IMV

Adjusted associations differed across the IMV outcomes (Table 2). Any IMV was associated with birth weight < 1000 g, gestational age ≤ 27 weeks, absence of spontaneous breathing, initial FiO_2_ > 0.30, and an initial set CPAP ≥ 12 cmH_2_O, whereas complete antenatal corticosteroid exposure was inversely associated. Early IMV was associated with gestational age ≤ 27 weeks, initial FiO_2_ > 0.30, and absence of spontaneous breathing. The latter produced a very large but imprecise estimate because 62 of 63 infants without spontaneous breathing received early IMV (aOR 151.93, 95% CI 18.26–1263.88). Initial set CPAP ≥ 12 cmH_2_O was not significantly associated with early IMV.

Among the 249 infants who did not receive early IMV, 59 (23.7%) subsequently received late IMV. All had spontaneous breathing at birth and received LISA; 44 (74.6%) had birth weight < 1000 g, 32 (54.2%) were born at ≤27 weeks, and 35 (59.3%) had an initial FiO_2_ > 0.30. Late IMV was associated with birth weight < 1000 g, initial FiO_2_ > 0.30, and birth in 2023 compared with 2021–2022. Initial set CPAP ≥ 12 cmH_2_O was not significantly associated with late IMV (Table 2).

Multicollinearity diagnostics did not indicate problematic collinearity. Variance inflation factors were 1.91 for birth weight < 1000 g and 2.00 for gestational age ≤ 27 weeks; all VIFs were <3, with a maximum value of 2.72.

### 3.3. Hospital Outcomes and Mortality by Gestational Age

Death before hospital discharge occurred in 117 of 381 infants (30.7%). BPD was recorded in 73 infants (19.2%), and the composite outcome of BPD or death occurred in 181 (47.5%). Mortality differed significantly across the original gestational-age categories (*p* < 0.001), reaching 83.3% among infants born at 22–24 weeks and generally decreasing at later gestational ages (Table 3).

### 3.4. Exploratory LISA Subgroup Analysis

Among 313 infants treated with LISA, 125 (39.9%) subsequently received IMV, 68 (21.7%) died before discharge, 62 (19.8%) had recorded BPD, and 122 (39.0%) experienced BPD or death.

Within the LISA subgroup, higher odds of IMV were associated with initial FiO_2_ > 0.30 (aOR 4.19, 95% CI 2.23–7.88; *p* < 0.001), birth weight <1000 g (aOR 2.55, 95% CI 1.23–5.27; *p* = 0.012), gestational age ≤27 weeks (aOR 2.37, 95% CI 1.21–4.66; *p* = 0.012), initial set CPAP ≥ 12 cmH_2_O (aOR 2.56, 95% CI 1.21–5.40; *p* = 0.014), and birth in 2023 compared with 2021–2022 (aOR 2.14, 95% CI 1.09–4.18; *p* = 0.027). The association with a complete antenatal corticosteroid course remained inverse but was not statistically significant (aOR 0.61, 95% CI 0.35–1.06; *p* = 0.080) (Supplementary Table S1). Given the exploratory nature of this analysis and the absence of adjustment for multiple comparisons, these findings should be interpreted as hypothesis-generating.

## 4. Discussion

In this cohort, early and late IMV showed different association patterns. Early IMV was associated primarily with lower gestational age, absence of spontaneous breathing, and higher initial FiO_2_, whereas late IMV was associated with extremely low birth weight and higher initial FiO_2_. Complete antenatal corticosteroid exposure was inversely associated with early IMV.

The early-IMV pattern is consistent with previous LISA and CPAP-failure studies in which lower gestational age, incomplete antenatal corticosteroid exposure, and greater initial respiratory-support requirements were associated with subsequent intubation [5,6,7,14,15]. Recent studies from Saudi Arabia and an LMIC tertiary-care setting in India similarly associated LISA failure with lower gestational maturity and early markers of clinical or respiratory severity [9,10], supporting the relevance of these factors outside established European neonatal networks. A prospective multicenter study identified an FiO_2_ value of approximately 0.29 during the second hour after birth as the optimal discriminator of CPAP failure [14]. Although numerically similar, our registry value was recorded during initial stabilization rather than at a predefined postnatal time point and therefore cannot be directly compared with the second-hour threshold reported in that study. FiO_2_ also depends on oxygen-saturation targets, interface leakage, CPAP delivery, and clinician practice and should therefore be interpreted as an early risk marker rather than an absolute indication for intubation.

Absence of spontaneous breathing should be interpreted primarily as a marker of immediate respiratory instability rather than as a precise independent predictor. Because 62 of 63 infants without spontaneous breathing received early IMV, the estimate was affected by near-complete separation. The wide confidence interval demonstrates substantial uncertainty, and the point estimate should not be used for individual risk prediction.

Respiratory drive is influenced by peripheral and central chemoreflex responses to hypoxemia, hypercapnia, and changes in pH [16]. However, the registry recorded only the presence or absence of spontaneous breathing and contained no physiological measures of chemoreflex function; therefore, these mechanisms could not be evaluated in this cohort.

The high prevalence of an initial set CPAP ≥ 12 cmH_2_O required particular consideration. Reassessment of the source data confirmed that this setting was recorded in 274 of 381 infants (71.9%) and reflected a local stabilization protocol describing a set CPAP of 15 cmH_2_O. However, recorded settings ranged from 8 to 15 cmH_2_O and use of ≥12 cmH_2_O declined across study periods, indicating that this setting was not applied uniformly. This differs from the initial CPAP range of 6–8 cmH_2_O recommended in current European guidance [1]. Although an initial set CPAP ≥ 12 cmH_2_O was associated with any IMV, it was not significantly associated with early or late IMV separately. Moreover, the registry documented the initial set CPAP, whereas delivered airway pressure and interface leakage were unavailable. The observed association therefore cannot be interpreted as an effect of higher delivered airway pressure and may instead reflect clinical severity, treatment escalation, local protocol characteristics, or changes in practice during the study period.

The inverse association between complete antenatal corticosteroid exposure and IMV is biologically plausible and consistent with evidence that antenatal corticosteroids reduce respiratory morbidity and neonatal mortality [17]. However, corticosteroid exposure was not randomized and may also indicate timely maternal admission, antenatal transfer, or better access to organized perinatal care. Likewise, the absence of an adjusted association between delayed cord clamping and IMV does not contradict evidence of its broader benefits after preterm birth [18].

Late IMV should be interpreted as a heterogeneous, timing-defined outcome rather than a distinct clinical phenotype. Although all infants in this group initially had spontaneous breathing and received LISA, they varied in maturity and initial oxygen requirement. Associations with extremely low birth weight, higher initial FiO_2_, and birth period should therefore be viewed as risk markers rather than evidence of specific mechanisms. Because the registry lacked the exact timing and indications for intubation and did not record intervening events such as sepsis, recurrent apnea, hemodynamically significant patent ductus arteriosus, pulmonary hemorrhage, or evolving lung disease, the mechanisms leading to late IMV could not be determined. Associations with birth period may reflect changes in referral patterns, survival, case mix, clinical practice, or documentation rather than an effect of calendar time itself. LISA can reduce exposure to endotracheal ventilation during surfactant administration but cannot eliminate subsequent IMV in infants with progressive respiratory failure [3,4,19].

Mortality was highest at the lowest gestational ages and generally decreased with advancing gestation. This finding is consistent with a large multicenter cohort study in the United States, which demonstrated significantly improved survival with increasing gestational age among extremely preterm infants [20]. However, direct comparison of mortality rates is limited by differences in eligibility criteria, active-treatment practices, case mix, and the denominators used to calculate survival. Associations between IMV and death or recorded BPD should not be interpreted causally because infants receiving IMV were generally more immature and clinically unstable, and the temporal sequence of deterioration, intubation, and subsequent complications was unavailable. The recorded BPD frequency of 19.2% and the BPD-or-death composite of 47.5% also require cautious interpretation because postmenstrual age at assessment and BPD severity were unavailable. These outcomes could not be reconstructed according to contemporary standardized definitions, limiting comparisons across centers and studies [21].

The strengths of this study include its two-center design, inclusion of infants across several gestational-age categories and birth periods, and separate evaluation of early and late IMV. The timing-specific analyses allowed clinically distinct phases of respiratory deterioration to be examined, while the use of routinely collected data provides evidence from an underrepresented Central Asian neonatal setting.

This study has several limitations. Its retrospective design is susceptible to residual confounding and confounding by indication. Analyses were restricted to 381 complete cases, and exclusion of eight infants may have introduced selection bias. The analytical dataset did not document standardized intubation criteria or patient-level indications for IMV, and serial information on respiratory effort, recurrent apnea, blood-gas parameters, oxygen-saturation trends, hemodynamic status, and response to surfactant therapy was unavailable. Consequently, we could not determine whether infants met uniform physiological criteria for CPAP failure or fully distinguish underlying illness severity from variation in clinical escalation decisions. Delivered airway pressure, interface leakage, serial FiO_2_, blood gases, and surfactant timing were also unavailable. Although the data originated from two centers, individual records did not contain a reliable center identifier; therefore, we could not account for clustering or adjust for between-center differences. Variation in case mix, referral patterns, respiratory management, and intubation thresholds between centers may have contributed to the observed associations, although the direction and magnitude of these effects could not be determined. Information on maternal conditions, mode of delivery, intrapartum distress, Apgar scores, retinopathy of prematurity, and necrotizing enterocolitis was incomplete and inconsistently recorded across the two registries and therefore could not be included in the analyses.

Prospective multicenter studies should apply standardized intubation criteria, retain reliable center identifiers, document the exact timing and indication for IMV, and measure delivered noninvasive pressure and time-dependent respiratory complications. Such data would allow more reliable evaluation of the pathways leading to early and late IMV.

## 5. Conclusions

In this retrospective cohort of very preterm infants, early and late IMV were associated with different clinical patterns. Early IMV was mainly associated with lower gestational age, absence of spontaneous breathing, and greater initial oxygen requirement, whereas late IMV was associated with extremely low birth weight and higher initial FiO_2_. Complete antenatal corticosteroid exposure was inversely associated with early IMV. The association between an initial set CPAP ≥ 12 cmH_2_O and any IMV should not be interpreted as a causal effect because it may reflect clinical severity and the local respiratory-support protocol. These findings may support early risk assessment but require prospective validation using standardized intubation criteria and detailed documentation of respiratory-support trajectories.

## Figures and Tables

**Table 1:** Perinatal and early respiratory characteristics according to timing of invasive mechanical ventilation.

Characteristic	Overall, *n* = 381	No IMV, *n* = 190	Early IMV, *n* = 132	Late IMV, *n* = 59	*p*-Value
Birth weight < 1000 g	241 (63.3)	85 (44.7)	112 (84.8)	44 (74.6)	<0.001
Gestational age ≤ 27 weeks	191 (50.1)	55 (28.9)	104 (78.8)	32 (54.2)	<0.001
Complete antenatal corticosteroid course	225 (59.1)	133 (70.0)	54 (40.9)	38 (64.4)	<0.001
Delayed cord clamping	328 (86.1)	178 (93.7)	93 (70.5)	57 (96.6)	<0.001
Multiple pregnancy	48 (12.6)	26 (13.7)	12 (9.1)	10 (16.9)	0.260
Conception by IVF	37 (9.7)	13 (6.8)	18 (13.6)	6 (10.2)	0.128
No spontaneous breathing at birth	63 (16.5)	1 (0.5)	62 (47.0)	0 (0.0)	<0.001
Initial FiO_2_ > 0.30	224 (58.8)	74 (38.9)	115 (87.1)	35 (59.3)	<0.001
Initial set CPAP ≥ 12 cmH_2_O	274 (71.9)	132 (69.5)	98 (74.2)	44 (74.6)	0.571
Caffeine administered	345 (90.6)	179 (94.2)	107 (81.1)	59 (100.0)	<0.001
LISA surfactant administration	313 (82.2)	188 (98.9)	66 (50.0)	59 (100.0)	<0.001

Note: IMV, invasive mechanical ventilation; CPAP, continuous positive airway pressure; FiO_2_, fraction of inspired oxygen; IVF, in vitro fertilization; LISA, less invasive surfactant administration.

**Table 2:** Adjusted associations of perinatal and early respiratory factors with any, early, and late invasive mechanical ventilation.

Variable	Any IMV, aOR (95% CI)	*p*-Value	Early IMV, aOR (95% CI)	*p*-Value	Late IMV, aOR (95% CI)	*p*-Value
Birth weight < 1000 g	2.85 (1.40–5.81)	0.004	1.55 (0.59–4.02)	0.373	3.51 (1.47–8.35)	0.005
Gestational age ≤ 27 weeks	2.40 (1.25–4.62)	0.009	3.91 (1.66–9.22)	0.002	1.31 (0.58–2.97)	0.510
Complete antenatal corticosteroid course	0.54 (0.31–0.93)	0.025	0.38 (0.20–0.72)	0.003	0.84 (0.42–1.68)	0.629
Delayed cord clamping	1.33 (0.47–3.71)	0.591	0.89 (0.30–2.64)	0.827	2.52 (0.46–13.77)	0.286
No spontaneous breathing at birth	40.22 (4.94–327.84)	<0.001	151.93 (18.26–1263.88)	<0.001	Not estimated	—
Initial FiO_2_ > 0.30	3.63 (1.97–6.71)	<0.001	4.39 (1.97–9.77)	<0.001	2.12 (1.02–4.39)	0.044
Initial set CPAP ≥ 12 cmH_2_O	2.33 (1.12–4.85)	0.023	2.09 (0.85–5.14)	0.108	2.11 (0.87–5.10)	0.097
Birth period: 2023 vs. 2021–2022	1.93 (0.98–3.80)	0.055	0.49 (0.21–1.17)	0.108	3.95 (1.68–9.26)	0.002
Birth period: 2024–2025 vs. 2021–2022	2.04 (0.83–5.03)	0.121	1.78 (0.61–5.13)	0.289	2.54 (0.75–8.58)	0.134

**Table 3:** Mortality before hospital discharge according to gestational age at birth.

Gestational Age, Weeks	Total, *n*	Deaths, *n* (%)	IMV, *n* (%)	No IMV, *n* (%)
22–24	30	25 (83.3)	28 (93.3)	2 (6.7)
25	32	19 (59.4)	26 (81.3)	6 (18.8)
26	71	27 (38.0)	51 (71.8)	20 (28.2)
27	58	15 (25.9)	31 (53.4)	27 (46.6)
28	68	9 (13.2)	25 (36.8)	43 (63.2)
29	68	14 (20.6)	20 (29.4)	48 (70.6)
30	54	8 (14.8)	10 (18.5)	44 (81.5)
Overall	381	117 (30.7)	191 (50.1)	190 (49.9)

Note: Mortality was defined as death before hospital discharge. The *p*-value for the comparison of mortality across gestational-age categories was <0.001 using Pearson’s chi-square test. IMV, invasive mechanical ventilation. Original gestational-age categories are presented descriptively in this table; the regression models used the prespecified categories of ≤27 and 28–30 weeks.

## Data Availability

The de-identified data supporting the findings of this study are available from the corresponding author upon reasonable request, subject to approval by the participating institutions and applicable ethical and data-protection requirements. The data are not publicly available because they contain potentially sensitive clinical information.

## Supplementary Materials

The following supporting information can be downloaded at: https://www.mdpi.com/article/10.3390/jcm15207879/s1, Table S1. Multivariable analysis of invasive mechanical ventilation among infants treated with LISA.
