# Supplementary material for: Perinatal and Early Respiratory Factors Associated with Invasive Mechanical Ventilation in Very Preterm Infants: A Retrospective Cohort Study from Kazakhstan
Source: J Clin Med. 2026 Oct 13;15(20):7879. doi: 10.3390/jcm15207879 (PMC13705521; doi:10.3390/jcm15207879)
Supplement: Supplementary file 1 [file jcm-15-07879-s001.zip › jcm-4565735-supplementary.pdf]

Supplementary materials

Supplementary Table S1. Multivariable analysis of invasive mechanical ventilation among infants treated with LISA

**Factor**	**Adjusted OR**	**95% CI**	**P value**
Birth weight <1000 g	2.55	1.23-5.27	0.012
Gestational age ≤27 weeks	2.37	1.21-4.66	0.012
Complete antenatal corticosteroid course	0.61	0.35-1.06	0.080
Delayed cord clamping	1.01	0.34-3.00	0.984
Initial FiO_2 >0.30	4.19	2.23-7.88	<0.001
Initial set CPAP pressure ≥12 cmH_2O	2.56	1.21-5.40	0.014
Birth period: 2023 vs 2021-2022	2.14	1.09-4.18	0.027
Birth period: 2024-2025 vs 2021-2022	1.54	0.60-3.91	0.368

Note: CI, confidence interval; IMV, invasive mechanical ventilation; LISA, lessinvasive surfactant administration; OR, odds ratio.
